# Convergent evolution on the hypoxia-inducible factor (HIF) pathway genes *EGLN1* and *EPAS1* in high-altitude ducks

**DOI:** 10.1038/s41437-018-0173-z

**Published:** 2019-01-10

**Authors:** Allie M. Graham, Kevin G. McCracken

**Affiliations:** 1 Department of Biology, University of Miami, Coral Gables, FL 33146 USA; 2 Rosenstiel School of Marine and Atmospheric Sciences, University of Miami, Miami, FL 33149 USA; 3 Human Genetics and Genomics, Hussman Institute for Human Genomics, University of Miami Miller School of Medicine, Miami, FL 33136 USA; 4 University of Alaska Museum and Institute of Arctic Biology, University of Alaska, Fairbanks, Fairbanks, AK 99775 USA; 5 Present Address: Department of Integrative Biology, Oregon State University, Corvallis, OR USA

**Keywords:** Evolutionary genetics, Evolutionary biology

## Abstract

During periods of reduced O_2_ supply, the most profound changes in gene expression are mediated by hypoxia-inducible factor (HIF) transcription factors that play a key role in cellular responses to low-O_2_ tension. Using target-enrichment sequencing, we tested whether variation in 26 genes in the HIF signaling pathway was associated with high altitude and therefore corresponding O_2_ availability in three duck species that colonized the Andes from ancestral low-altitude habitats in South America. We found strong support for convergent evolution in the case of two of the three duck species with the same genes (*EGLN1, EPAS1*), and even the same exons (exon 12, *EPAS1*), exhibiting extreme outliers with a high probability of directional selection in the high-altitude populations. These results mirror patterns of adaptation seen in human populations, which showed mutations in *EPAS1*, and transcriptional regulation differences in *EGLN1*, causing changes in downstream target transactivation, associated with a blunted hypoxic response.

## Introduction

**Fig. 1:**
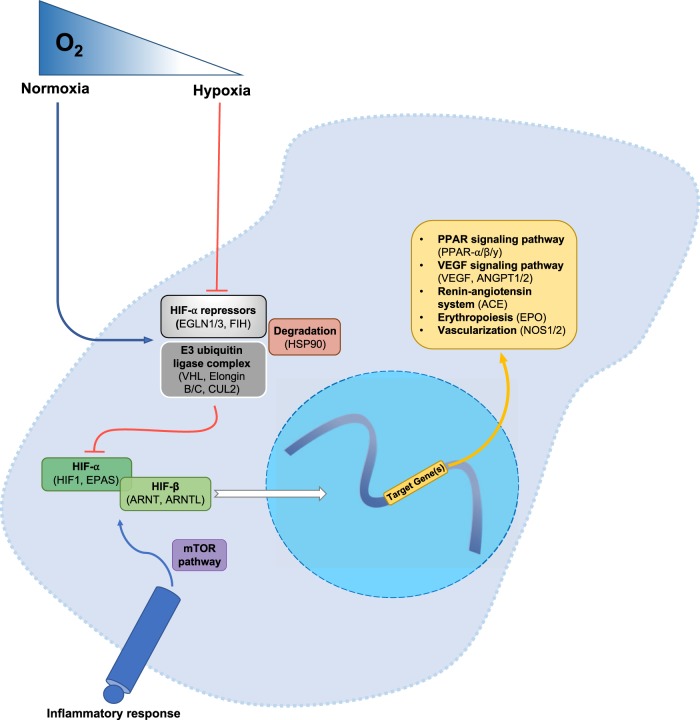
A simplified version of the HIF pathway in a cell, with a focus on specific aspects (repressor complexes, HIF transcription factors, downstream target genes) included in the 26 genes assayed (also see Supp. Fig. 1; Kanehisa & Goto, 2000)

Convergent evolution of adaptive traits in distantly related organisms under the same selective pressures is common, at the genomic, cellular, and phenotypic level (Conte et al. 2012; Gompel and Prud’homme 2009; Losos 2011; Stern and Orgogozo 2009). There are multiple levels, at which the underlying genetic mechanisms for adaptive traits can occur, including different genes in the same pathway, gene, functional region, amino acid, or nucleotide (Storz 2016). Hypoxia (or low O_2_) is one selective pressure that stimulates a similar physiological response across metazoans allowing organisms to match O_2_ supply and demand (Semenza 2007b). During reduced O_2_ supply, changes in gene expression are mediated by a specific transcription factor family that is considered a “master regulator” of O_2_ homeostasis, known as hypoxia-inducible factors (HIF) (Lisy and Peet 2008; Semenza 2007a; Webb et al. 2009). In mammalian cells, HIF transcription activity is regulated post-translationally through degradation under normoxic conditions. However, under hypoxic conditions, such degradation of the HIFA (*HIF1α, HIF2α/EPAS1*) subunits does not occur, therefore allowing the HIF heterodimers to enter the nucleus where they recognize hypoxia-response elements (HREs) within the promoters of a large number of genes, effecting changes in transcriptional activity (Fig. 1) (Wenger et al. 2005). Many of these target genes increase O_2_ transport to hypoxic tissues by promoting red blood cell maturation and angiogenesis/vasomotor control (Haase 2013; Majmundar et al. 2010).

Therefore, it is not surprising that in many organisms, HIF transcription factors, and a number of their downstream targets, have been implicated in adaptation to high-altitude environments (Hanaoka et al. 2012; Li et al. 2014; Qiu et al. 2012; Qu et al. 2013; Wang et al. 2014), as well as in other O_2_ depleted environments (Aggarwal et al. 2010; Ge et al. 2012; Peng et al. 2011; Rytkönen et al. 2007; Terova et al. 2008; Xing et al. 2013). Most examples of convergent evolution in the HIF pathway have been documented in high-altitude human populations (Scheinfeldt and Tishkoff 2010; Simonson et al. 2012). In Andean people, *VEGF*, *NOS2A*, and *EGLN1* are the strongest-supported candidates (Bigham et al. 2010; Bigham et al. 2009), whereas in Tibetans, *EPAS1* and *EGLN1* have been identified (Beall et al. 2010; Jeong et al. 2014; Lorenzo et al. 2014; Peng et al. 2011; Simonson et al. 2012; Simonson et al. 2010; Xu et al. 2011; Yi et al. 2010). However, studies focused on Ethiopian highlanders suggest the involvement of a different combination of HIF genes (Alkorta-Aranburu et al. 2012; Huerta-Sánchez et al. 2013; Scheinfeldt et al. 2012).

High-altitude species offer an unparalleled opportunity to understand the molecular and physiological bases of adaptation to hypoxic environments. Therefore, because of the degree to which the HIF pathway is a frequent target of selection for adaptation to hypoxia, we tested whether genetic changes in various parts of the HIF signaling pathway might be statistically associated with high-altitude adaptations in Andean waterfowl species.

### Study organisms

Our study includes three species that have low-altitude populations, in addition to high-altitude populations that are continuous year-round residents at > 4000 meters; at this altitude, the O_2_ partial pressure (pO_2_) is ~ 60% of that at sea level. These include the yellow-billed pintail (*Anas georgica)*, cinnamon teal (*Anas cyanoptera)*, and the speckled teal (*Anas flavirostris flavirostris/Anas f. oxyptera*), which were the focus of previous studies examining adaptive changes in hemoglobin (Hb) function (McCracken et al. 2009a; McCracken et al. 2009b; McCracken et al. 2009c; Natarajan et al. 2015). The benefits of using these species is that each of these duck lineages are distantly related by several millions of years of divergence (Johnson and Sorenson 1999) and each species independently colonized the same high-altitude wetlands and puna grasslands of the Andean Plateau (Altiplano), with ancestral low-altitude populations colonizing high-altitude regions and not the reverse (Fjeldså 1985; McCracken et al. 2009a). Although the overall genomic differentiation between populations is still very low for each species (e.g., *F*_*ST*_ < 0.06), Hb differentiation was markedly higher, with *F*_*ST*_ ranging from 0.89 to 1.00 for the amino-acid polymorphisms that have been experimentally validated in influencing Hb–O_2_-binding affinity (Natarajan et al. 2015). This suggests that even though historical gene flow has been relatively high for each of these species, selection on genes related to high-altitude function have led to strong patterns of differentiation, producing unambiguous genomic outliers with measurable functional effects. Generally speaking, this type of pattern occurs through the process of migration-selection balance, when the strength of selection is strong enough to overcome homogenizing forces of gene flow, i.e., if the selection coefficient *s* is much greater than the migration rate *m* (Charlesworth et al. 1997; Slatkin 1987; Yeaman and Whitlock 2011). These three Andean duck species are thus ideal for assessing targets of local adaptation in the HIF signaling pathway through outlier analyses, i.e., genomic scans.

### Objectives

Here, using target-enrichment sequencing, we test whether variation in 26 genes in the HIF signaling pathway is associated with high altitude in three duck species that colonized the Andes from ancestral low-altitude habitats in South America. Analysis of these three species allowed us to identify whether (a) polymorphism in HIF pathway genes is statistically associated with occupancy of high-altitude hypoxic environments, and (b) if so, whether the same genes functional region, amino acids, or nucleotide substitutions converged in each duck lineage. Here, we focus on the role of exonic variation (i.e., nonsynonymous changes) owing to our ability to better extrapolate its ability to have a direct physiological effect, though we do briefly discuss the potential for intronic variation to also affect a functional change. Ultimately, we found strong support for convergent evolution on the HIF pathway (*EPAS1, EGLN1*), with the same exonic regions of *EPAS1*, and intronic regions of *EGLN1*, exhibiting sharply demarcated outliers with a high probability of directional selection in high-altitude populations of two of the three duck species.

## Materials and methods

### Specimen collection

A total of 60 individuals were used for this study from three different Andean duck species. For the cinnamon teal, individuals from low-altitude populations are the *A. c. cyanoptera* subspecies (*n* = 10; elevation range 8–23 m) and from high-altitude are the *A. c. orinomus* subspecies (*n* = 10; elevation range 3533–3871 m) (Wilson et al. 2013). For the speckled teal, individuals from low-altitude populations are the *A. f. flavirostris* subspecies (*n* *=* 10; elevation range 77–860 m) and from high-altitude are the *A. f. oxyptera* subspecies (*n* = 10; elevation range 3211–4405). For the yellow-billed pintail, individuals from both populations are taxonomically identified as *Anas georgica spinicauda*. A total of 20 yellow-billed pintails were collected from low- (*n* = 10; elevation range 292–914 m) and high-altitude (*n* = 10; elevation range 3332–4070 m). Additional information regarding the samples, including location and dates collected, have been previously described in Graham et al. (2018) and McCracken et al. (2009b).

More detailed information about the phylogeography of these three species, including their population sizes, dispersal behavior across elevational gradients, and gene flow is available in (Wilson et al. 2013), (Graham et al. 2018), and (McCracken et al. 2009a; McCracken et al. 2009c), respectively.

Although these relatively small sample sizes make it impossible to demarcate genomic outliers with low *F*_*ST*_ such as those experiencing weaker selection, our previous research has shown that these samples can successfully be used to identify extreme outliers with high *F*_*ST*_ demonstrated to have large phenotypic effects (Natarajan et al. 2015).

### HIF pathway gene rationale

HIF pathway genes were chosen based on a combination of (1) being found to be a gene of interest in previous studies of humans using whole-genome scans looking to identify genetic adaptations to high-altitude or hypoxic environments, (2) being a part of the canonical HIF pathway, either through being a known downstream target, or a part of the repression machinery of the pathway, and/or (3) being a part of similar transcription factor families (i.e., bHLH-PAS containing proteins).

A total of 26 genes were able to have probes created for them (Fig. 1, Supp. Fig. 1, Supp. Table 1). These genes represent a wide-breath of coverage across the various parts of the HIF pathway (upstream players, major Transcription Factors/TF, TF repressors, and downstream targets). We were able to capture 100% of the major TFs, and 4/5 of the E3 ubiquitin ligase complex associated with HIF repression; we are unable to capture all known downstream targets of the HIF complex owing to the sheer number of targets it binds to, though we were able to capture all relevant genes in that category (i.e., those which had been major outliers in other high-altitude organisms). Ultimately, we were able to capture a vast majority of the pertinent elements of the HIF pathway incorporating 26 genes, most of which possessed long-coding regions.

Of particular interest were the major HIF pathway TFs (*HIF1a*, *HIF2a*/*EPAS1*); these genes had prior expectations for showing signatures of selection (see ‘Identification of Outlier Loci’ section in the Methods). All HIFα proteins are characterized by the presence of an N-terminal bHLH DNA-binding domain upstream of two PAS domains. In addition, all vertebrate α-subunits include an inhibitory domain called the O_2_-dependent degradation domain, and an N-terminal transactivation domain (NTAD) (Graham and Presnell 2017). *HIF1A* and *EPAS1* proteins are characterized by the presence of a C-terminal transactivation domain (CTAD) located at the C-terminal end of the protein (Lisy and Peet 2008). These domains are considered critical to the overall function of HIF proteins: the bHLH domain contacts the core nucleotides of HIF-responsive elements (Dinkel et al. 2015), whereas bHLH and PAS domains together mediate both dimerization and sequence specific DNA-binding (Crews and Fan 1999; Ledent and Vervoort 2001). The NTAD is thought to confer target specificity (Hu et al. 2007), whereas the CTAD is required for full HIF activity (Lando et al. 2002) and interactions with co-activators (Carrero et al. 2000; Ema et al. 1999).

### DNA extraction and target-enrichment sequencing

Genomic DNA was extracted from muscle tissue using a DNeasy Tissue Kit (Qiagen, Valencia, California, USA) following manufacturer's protocols. In-solution target capture was used to selectively enrich libraries for regions of interest prior to sequencing (Gnirke et al. 2009). All steps of the process were performed by MYcroarray (Ann Arbor, MI), now Arbor Biosciences. A custom MYbaits® biotinylated ssRNA target capture baitset was designed for enriching target sequences. In total, 26 genes associated with the HIF pathway from the duck nuclear genome were selected from the Ensembl *A. platyrhynchos* v1.0 genome. These 26 sequences were first screened using the web server version of RepeatMasker (http://www.repeatmasker.org/cgi-bin/WEBRepeatMasker) with default settings and selecting *A. platyrhynchos* as the species, soft-masking all repeats. Next, 120mer probes at 2× tiling density were designed from these soft-masked sequences. All candidate probes were then screened against the duck genome using MYcroarray’s in silico bait analysis software pipeline, in order to filter out any probes that were non-specific in the genome. A final set of 12,062 filtered probes was chosen for 26 genes (Supp. Table 1), which included all probes that (a) did not contain any soft-masked regions and (b) passed MYcroarray’s most liberal filtering threshold. Following hybridization, target regions were purified on magnetic beads followed by post-hybridization amplification to ligate indexing sequences. Sequencing was performed on an Illumina HiSeq platform paired-end (100 bp) with a 250–300 bp insert size.

### RAD-seq data

Previously generated RAD-Seq (Restriction Site Associated DNA Sequencing) data were utilized in order to function as a “backdrop” i.e., genomic background, for analyses for the HIF pathway enriched SNP data; the RAD-seq were generated using methods described in (Natarajan et al. 2015) and (Graham et al. 2018). All RAD clusters were then subjected to a BLAST search (database—nr, *e* value < e^−10^, annotation cutoff > 50) in Blast2GO (Conesa et al. 2005) using the taxonomy filter for birds (taxa: 8782, Aves) to determine gene identity. Ultimately, SNPs on clusters whose BLAST result was that of a protein-coding gene was then combined with the newly generated target-enrichment data in order to perform selection analyses.

### Target enrichment/HIF pathway sequence assembly pipeline

Sequences from the target-enrichment sequencing were received pre-parsed by individual, with adapters trimmed and quality filtered (*Q* < 30). Additional adapter trimming was performed utilizing *fastq-clipper* (AGATCGGAAGAGC) and remaining sequences were then filtered by length and quality using *fastq*-quality filter (reads < 20 bp, and *Q* < 30) from the FASTX-Toolkit v. 0.0.13 package (Gordon and Hannon 2010). A custom pipeline (scripts located at https://github.com/amgraham07) was created to remove orphan sequences and assemble sequences against the 26 reference genes using BWA v0.7.15 (Li and Durbin 2009). The Samtools package v1.3.1, including BCFtools v.1.3.1 (Li et al. 2009) was then used to create a VCF file, as well as provide assembly statistics (i.e., bp-by-bp coverage). These programs used in the pipeline called SNP variants that were variable against the mallard genome reference, including indels (insertion/deletion); however, the indel information was excluded in the final data set, as the software used does not accommodate indels.

### Identification of outlier loci

A combined data set used for detection of outlier loci contained (a) all SNPs associated with the HIF pathway from the target-enrichment sequencing data and (b) SNPs from RAD clusters whose BLAST results hit back to a protein-coding gene (see previous section). The latter provides both a comparison of a priori candidate genes and a genome-wide reference data set for the potential HIF outliers to be analyzed. Ultimately, any outliers described in the results are ones associated with the HIF pathway from target-enrichment sequencing, as the RAD-seq data set only serves as a “backdrop” for analysis.

We tested for signatures of directional selection using three different outlier detection techniques, which minimized the risk of detecting false positives. Outlier loci were considered to be those that were identified by at least two of the three methods as being significant outliers, with the most significant outliers classified as being identified by all three methods.

First, a genomic scan was performed by obtaining pairwise site-by-site *F*_*ST*_ calculated in Arlequin v. 3.5 (Excoffier and Lischer 2010); these values were then imported into JMP Pro 12 for distribution visualization, as well as for percentile calculations for each species. Candidate loci *F*_*ST*_ values that exceeded the 99th percentile of the *F*_*ST*_ values were therefore considered likely targets of selection. Second, MCHEZA, which implements the Dfdist function, was used to demarcate markers putatively under positive directional selection (Antao and Beaumont 2011). MCHEZA analyses were based on the Infinite Alleles Model with 50,000 simulations, a confidence interval of 0.95 and a false-discovery rate of 0.01, using the neutral mean *F*_*ST*_ and forcing mean *F*_*ST*_ options. It is important to note here, that MCHEZA was used instead of LOSITAN (Antao et al. 2008) owing to the large size of the data set, and current issues with Java updates (T. Antao, per comm.).

Third, a Bayesian approach as implemented in BayeScan v. 2.1 was used to again identify putatively selected loci. BayeScan uses a logistic regression model to separate locus-specific effects of selection from demographic differences (Foll and Gaggiotti 2008). For each SNP, BayeScan estimates the posterior distribution under neutrality *α* = 0 and separately allowing for selection *α* ≠ 0 and computes the posterior odds ratio (PO) as a measure of support for the model of local adaptation relative to neutral demography. Foll (2012) proposed a logarithmic scale for the posterior odds defined as: Bayes Factor (BF) 3–10 substantial (log_10_PO > 0.5–1.0); BF 10–32 strong (log_10_PO > 1–1.5); BF 32–100 very strong (log_10_PO > 1.5–2.0); and BF 100–∞ decisive (log_10_PO > 2.0–∞) evidence for accepting a model. In the genome scans, a threshold for log_10_PO > 0.5 (substantial), representing a Bayes Factor of 3.0 and posterior probability of 0.76, was used for a marker to be considered under selection. Therefore, loci identified as “substantial” probability of being under selection were classified as being significant outliers, under this approach.

## Results

### HIF pathway assembly information and population divergence estimates

The total number of bp for the 26 genes in the target-enrichment data set was 839,657 bp per individual, with the goal of ~ 50× coverage per gene in each individual (Supp. Tables 1 and 2). Across all individuals of the three species, the final coverage for each gene was between 74 × (*P4HA3*) and 584 × (*CUL2*), with an average across all genes of 414×. This coverage included genes whose total regions covered 81–100% of the reference mallard sequence, with an average of 98.1% ( ± 3.9%) (Supp. Table 2). Within each species, there were a total of 16,339 HIF pathway SNPs for the cinnamon teal, 21,674 SNPs for speckled teal, and 26,484 SNPs for the yellow-billed pintail, which likely reflects apparent differences in the effective population sizes (*Ne*) of these three species (Graham et al. 2018; McCracken et al. 2009c; Wilson et al. 2013).

The RAD-Seq yielded a total of 18,145 SNPs from cinnamon teal, 47,731 SNPs from speckled teal, and 49,670 SNPs for the yellow-billed pintail (See Natarajan et al. 2015) for other summary statistics concerning the RAD-seq data sets). Among these, 2762 SNPs for cinnamon teal, 6280 SNPs for speckled teal, and 6523 SNPs for yellow-billed pintail mapped to a gene region, representing 13–15% of the total RAD-Seq clusters. Of the subset of those RAD-seq clusters that mapped to a gene, 1441 clusters with an average of 1.9 SNPs per cluster for the cinnamon teal at an average of 121× coverage, 2600 clusters with an average of 2.4 SNPs per cluster for the speckled teal at an average of 85× coverage, and 2619 clusters with an average of 2.5 SNPs per cluster for the yellow-billed pintail at an average of 112× coverage.

Initially, *F*_*ST*_ was calculated for three separate subsets of the data between each pair of low- and high- altitude populations: (1) target-enrichment/HIF pathway only, (2) RAD-Seq gene-only, and from the (3) combined HIF pathway and RAD-Seq gene-only data sets. All species showed similar average *F*_*ST*_ values, except for the HIF pathway, which had slightly greater overall divergence than the RAD-Seq data set. These estimates mirrored previous calculations with nuclear loci in these species (Table 1) (McCracken et al. 2009a; McCracken et al. 2009c; Wilson et al. 2013).

**Table 1:** Estimates of divergence (*F*_*S****T***_) for the three species across the different data sets, including (1) the variants associated with the HIF pathway (i.e., 26 genes), (2) variants associated with the RAD-seq data set whose sequence had a significant BLAST hit to any protein-coding gene, and (3) the combined variant data set including both (1 and 2)

	*F*_*ST*_		
Species	HIF pathway enriched	RAD-seq (genes only)	Combined
Cinnamon teal	0.051	0.042	0.04
Yellow-billed pintail	0.045	0.013	0.02
Speckled teal	0.124	0.058	0.06

### Identifying HIF pathway gene regions with high F_ST_

HIF pathway gene regions with high *F*_*ST*_ were examined using three different methods (percentile, Dfdist, Bayesian) for cross-comparison among species. These analyses were performed with a combined data set of SNPs from both the HIF pathway enriched sequence data, as well as the RAD-seq gene-only sequence data.

There was general evidence of convergent evolution for the HIF pathway across high-altitude populations of all three duck species (Supp. Table 3–5). For cinnamon teal, the results from the Dfdist analyses showed seven genes with outliers, with *EPAS1* (36 SNPs) and *NOS1* (10 SNPs) having the most hits, followed by *EGLN1* (4 SNPs), *CLOCK* (2 SNPs), and *PPARA1*, *MTOR*, and *P4HA3* (one SNP each); however, none were significant in the corresponding Bayesian analysis.

**Fig. 2:**
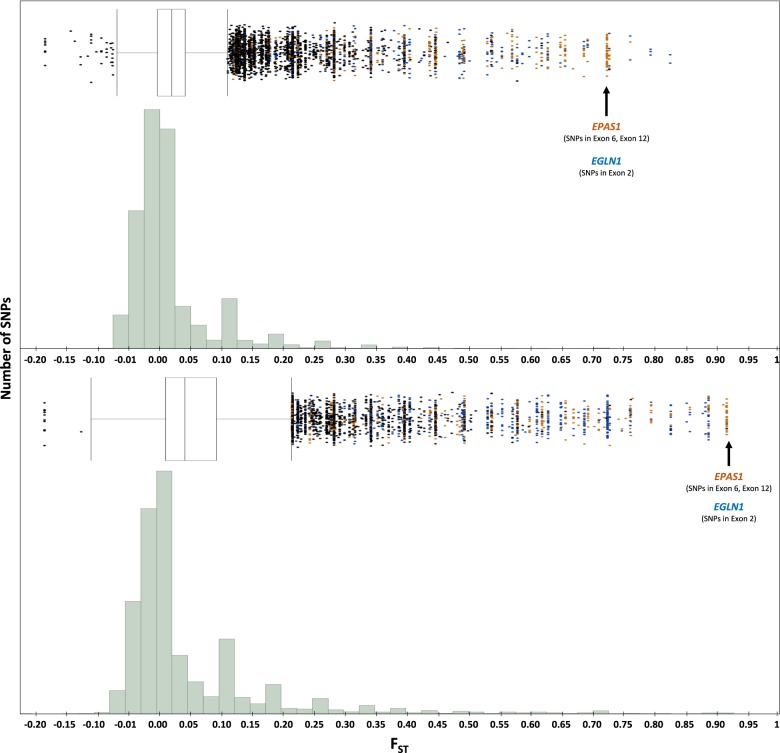
RAD-seq cluster distribution against a measurement of population divergence for yellow-billed pintail (top) and speckled teal (bottom): histogram is distribution of all SNP variants (RAD-seq, and HIF pathway genes), with specific genes of interest highlighted: *EGLN1* (blue) and *EPAS1* (orange), whereas markers with black arrows signify the nonsynonymous/synonymous amino-acid variants in exonic regions of *EGLN1* and *EPAS1*

**Fig. 3:**
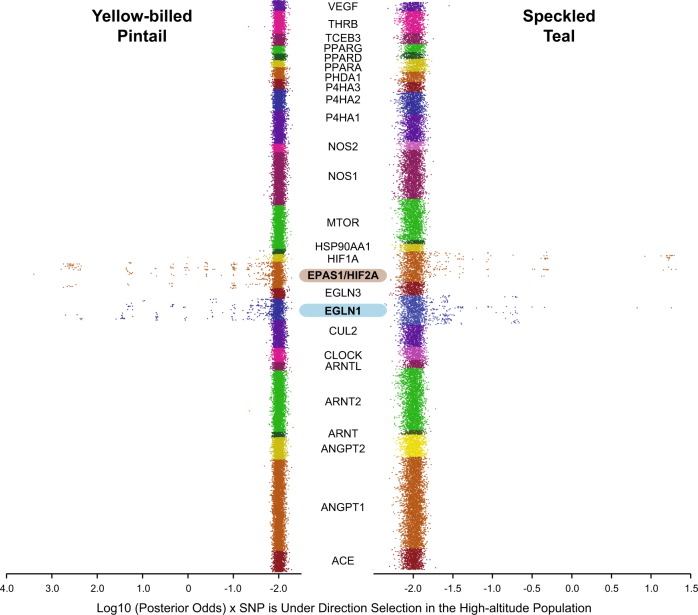
Manhattan scatterplots of each of the 26 HIF pathway gene members listed in alphabetical order, and with their positions in numerical order for both yellow-billed pintail and speckled teal. The two genes with any significant outliers are highlighted (log_10_PO > 0.5; *EGLN1*, *EPAS1*)

**Fig. 4:**
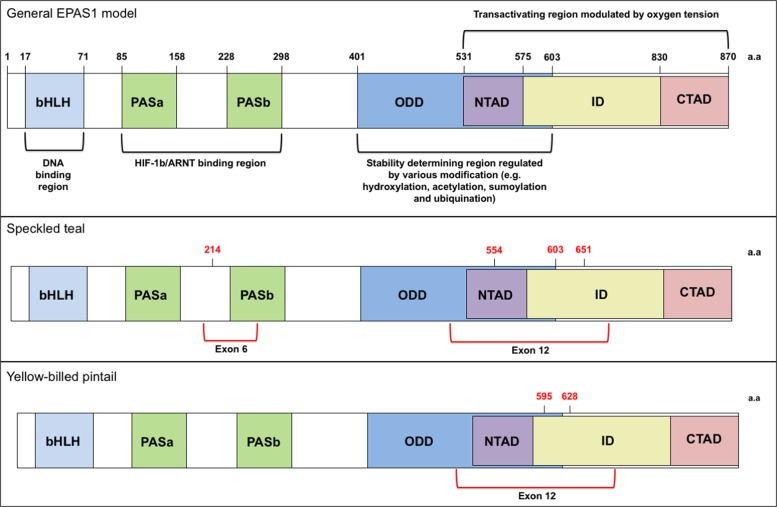
General protein model of *EPAS1*, modified from (Hong et al. 2004), including the canonical domains of the HIF gene family. The variants associated with exonic regions in the gene for both yellow-billed pintail and speckled teal are shown with their associated location/proximity to those domains

The strongest outliers in both speckled teal and yellow-billed pintail were *EGLN1* and *EPAS1* and were highly significant in both the Bayesian and Dfdist analyses for both species (Figs 1 and 2, Supp. Table 4, 5). For the HIF pathway SNPs, although most of the SNPs associated with these two genes were located in intronic/noncoding regions, both speckled teal and yellow-billed pintail contained highly significant *F*_*ST*_ outliers in various exonic regions of *EPAS1* and *EGLN1*—specifically, exon 6 (YBP) and exon 12 (ST, YBP) of *EPAS1*, and in exon 2 (YBP) of *EGLN1* (Figs 3 and 4; Supp. Fig. 2). All exonic SNP variants of particular interest in the low-altitude populations were identical to the presumably ancestral alleles found in the mallard reference, whereas the SNP variants in high-altitude populations were derived. None of the outliers discussed were fixed (i.e., *F*_*ST*_ = 1.0) between high- and low-altitude populations for any of the three species.

### Conservation of exons in human and mallard EPAS1 and EGLN1

Further analyses focused on those high *F*_*ST*_ variants found in exon 6 and 12 in *EPAS1* and exon 2 in *EGLN1*. We compared mallard with human in an explicit test to look for conservation of exon structure. If these exons were not the same, then we could not realistically draw any comparisons from previous literature between the two exons in terms of their potential function. In order to compare the similarity of exon sequence between ducks and humans, the full protein sequences from mallard and human were aligned to each other and exons 6 and 12 were annotated: *EPAS1* (human, Q99814; mallard U31HW4), and *EGLN1* (human Q9GZT9; mallard, U3J106). Overall, the alignment showed sufficient conservation between sequences, in combination with orthology quality-control statistics for the whole gene from ENSEMBL (GOC—100/100; WGA–91.92/100).

In speckled teal and yellow-billed pintail, four out of the five SNPs resulted in nonsynonymous substitutions in the first and second codon positions (Supp. Fig. 2). Specifically, in EPAS1 there was a Cys → Tyr substitution at amino-acid position 23 in exon 6 and then Arg → His and Tyr → His substitutions at amino-acid positions 32 and 127, respectively, in speckled teal. In yellow-billed pintail, there were two nonsynonymous substitutions at two different positions in exon 12, Pro → Glu at position 71 and Ala → Thr at amino-acid position 104 (Table 2). For *EGLN1*, the same general approach was taken, by comparing the mallard and human protein sequence, and human and mallard nucleotide sequence for exon 2; however, the observed nucleotide change resulted in a synonymous substitution.

**Table 2:** Nonsynonymous changes to exon 12 in *EPAS* of the speckled teal (ST) and yellow-billed pintail (YBP) between their respective high- and low-altitude populations (also see Fig. 3)

Species	Low	High	Within exon aa position	Whole protein aa position
ST	Cys	Tyr	26 (of exon 6)	214
ST	Arg	His	32 (of exon 12)	554
ST	Tyr	His	126 (of exon 12)	651
YBP	Pro	Glu	71 (of exon 12)	595
YBP	Ala	Thr	104 (of exon 12)	628

### Biochemical properties and structural locations of amino-acid replacements in EPAS1

The two nonsynonymous SNPs in *EPAS1* exon 12 of yellow-billed pintail resulted in substitutions of amino acids with very different biochemical properties. The Pro → Glu substitution at amino-acid position 71 resulted in a change from a neutral, polar amino acid, to a hydrophobic, nonpolar amino acid in the high-altitude population. The Ala → Thr substitution at amino-acid position 104 resulted in a change from a nonpolar amino acid with a hydrophobic side chain, to a neutral, polar amino acid that has a non-aromatic hydroxyl group attached in the high-altitude population (Supp. Fig. 2).

Of the three nonsynonymous SNPs in *EPAS1* exon 12 in speckled teal two resulted in conservative substitutions with similar biochemical properties, Cys → Tyr at position 23 and Arg → His at position 32. Though the Tyr → His at amino-acid position 127, resulted in substitution of a nonpolar aromatic amino acid with a hydrophobic side chain, to a basic, polar amino acid with a positively charged side-chain in the high-altitude population.

Locations of these variants were unable to be placed on existing Protein Data Bank protein models, owing to all available current models containing only the bHLH, and PAS domains, thus lacking any of the ODD/NTAD/CTAD domain regions that are characteristic of the HIF transcription factors (Graham and Presnell 2017). This makes potential functional assessment of the variants in the future problematic.

## Discussion

The results of this study show a strong degree of convergent evolution on the HIF pathway in two of the three Andean duck species assayed in this study. Although the cinnamon teal showed evidence of outliers in seven HIF pathway genes, none were significant in the corresponding Bayesian analysis; the evidence for convergence on the HIF pathway as a means of adaptation to high-altitude environments was much stronger in the speckled teal and yellow-billed pintail—specifically, our results suggest that selection acted on the same genes (*EPAS1, EGLN1;* Figs 2 and 3) and even the same gene regions (exon 12 in *EGLN1*), though not at the same amino acid positions (Fig. 4; Supp. Fig. 2). It is important to note that all SNP variants for the two species revealed the low-altitude populations being identical to the “ancestral” alleles found in the mallard reference, thus suggesting that such variants are potentially adaptive in the high-altitude populations only.

We focused the results and the discussion more heavily toward those nonsynonymous variants with protein-coding regions because of what prior work has shown regarding those regions (exon 6, and 12 in *EPAS1*); however, we fully acknowledge the potential for intronic variants (as has been found in human populations) to affect the regulation of gene activity (Hsiao et al. 2016; Park et al. 2018), though it is difficult for us to infer such functions from the sequence data presented in this study. Ultimately, our results also show of convergence with previously studied populations of other organisms living at high-altitudes, specifically humans (which have also shown *EPAS1* and *EGLN1* as major outliers), extending to the same genes associated with adaptations to low-O_2_ environments.

### The role of the HIF pathway in adaptations to high-altitude environments

Members of the HIF pathway are consistently among the top genomic outliers in organisms dealing with chronic hypoxic stress, and is especially true for the major transcription factor machinery (*EPAS1*) and its repression machinery (*EGLN1*). These genes are frequently targets for selection, because genes that form a hub in a regulatory network between a series of upstream activators and a battery of downstream effector genes are thought to be more likely to become the targets of repeated parallel or convergent evolution (i.e., “hotspot” genes) (Martin and Orgogozo 2013; Rosenblum et al. 2014; Stern and Orgogozo 2009). In addition, such variation within the gene would be more likely to occur in exonic regions/domains that are associated with protein–protein interactions rather than DNA-binding activity (Wagner and Lynch 2008); for example, if they play an essential role in coordinating the expression of target genes in response to multiple input signals in a way that cis-regulatory elements cannot (Wagner and Lynch 2010). Both *EPAS1* and *EGLN1* fit these criteria because they (1) sit at important junction which ultimately regulates the physiological response through a wide array of downstream targets, and (2) have shown causal variants within protein–protein interaction domains (i.e., ODD/NTAD/CTAD); therefore, these genes are prime candidates to be labeled as “hotspot” genes.

Much of what is known about how the HIF pathway is associated with adaptation to high-altitude environments comes from early genome-wide association studies in humans; these genome-wide scans identified many candidate genes that may contribute to adaptive evolution, including two genes (*EPAS1* and *EGLN1*) that are involved in the HIF pathway, which showed the strongest signals of selective sweeps in Tibetan and Andean humans (Beall et al. 2010; Bigham et al. 2010; Peng et al. 2011; Simonson et al. 2012; Xu et al. 2011), as well as in other high-altitude human populations such as Caucasian/Russians (Pagani et al. 2012), Mongolians (Xing et al. 2013) and others populations in the Himalayas (Arciero et al. 2018; Hackinger et al. 2016). In addition, animals residing in similar high-altitude environments have also had the same HIF pathway members identified as major outliers, including ungulates (Song et al. 2016), birds (Qu et al. 2013), pigs (Ai et al. 2014; Li et al. 2013), and dogs (Gou et al. 2014; Li et al. 2014).

The function of some of these variants has only recently been assessed; these variants are generally associated with the relationship between *EPAS1* and *EGLN1*, so it is important to understand dynamics between the transcription factor (*EPAS1*) and its repressor (*EGLN1*). In most organisms, under normoxia, *EGLN1* can perform its O_2_-dependent hydroxylase function on both *HIF1A* and *EPAS1* proteins, which triggers degradation via additional enzymatic complexes. However, under hypoxia, the hydroxylase activity of *EGLN1* is suppressed, resulting in the accumulation of HIFAs that can then activate hundreds of downstream target genes, thus inducing numerous physiological responses, including changes in red blood cell (RBC) production (Haase 2013; Hu et al. 2003).

In high-altitude human populations, variants have been shown to alter this relationship. Specifically, *EGLN1* has been shown to have both loss-of-function, and gain-of-function mutations, depending on the study population (Song et al. 2014; Xiang et al. 2013). Specifically, these mutations have been shown to affect the ability of *EGLN1* to target *EPAS1* for further degradation, ultimately activating a broad range of effects orchestrating acclimatization to hypoxia, and potentially leading to a blunted hypoxic response (Lorenzo et al. 2014; Song et al. 2014; Xiang et al. 2013). Unlike *EGLN1* variants, many *EPAS1* variants are located in the noncoding regions, suggesting that they could affect the regulation of *EPAS1* at the transcriptional level (Peng et al. 2017; Peng et al. 2011).

### EPAS1 and EGLN1 in high-altitude adaptation in Andean ducks

Functional work on the role of the *EPAS1* and *EGLN1* variants associated with high-altitude adaptation in humans is the best studied (see previous section); in addition, these genes are highly conserved in nucleotide composition and exon structure across vertebrates, including between ducks and humans (Supp. Fig. 3). Such information is crucial because it allows us to extrapolate from information on well-studied organisms (i.e., humans) to ones that have less information available (i.e., ducks).

Although our results identified no variation in coding regions of *EGLN1*, the variation found within intronic regions of the high-altitude duck species could still suggest a role for transcriptional regulation of *EGLN1* in high-altitude adaptation in the Andean duck species of this study; however, it is hard to hypothesize further about its potential effect in the context of these duck species, beyond speculating. Unlike *EGLN1* in our study, we found that *EPAS1* contained outliers located within exons: four in speckled teal (one synonymous and three nonsynonymous) and two in yellow-billed pintail (both nonsynonymous; Supp. Table 3, 4). The nonsynonymous variants are located in two specific exons, both of which make-up parts of critical domains of the *EPAS1* protein that define HIF function, i.e., the PAS domain (exon 6) and ODD/NTAD domain (exon 12; Fig. 4); therefore, we can hypothesize more thoroughly about possible functional effects on physiological changes that would be associated with adaptation to high-altitude. Specifically, the PAS domains mediate both dimerization and sequence-specific DNA binding (Crews and Fan 1999; Ledent and Vervoort 2001), whereas the ODD is the target of HIF repression machinery during normoxia (i.e., oxygen-dependent degradation), and NTAD is thought to confer target specificity (Hu et al. 2007).

Interestingly, variants in the same exons we identified in *EPAS1* (exon 6, exon 12) have also been implicated in being associated with various physiological responses to oxygen stress in humans and other organisms (Buroker et al. 2012; Gale et al. 2008; Newman et al. 2015; Percy et al. 2008a; Percy et al. 2008b; Yi et al. 2010); however, exon structure is not always conserved, owing to exon shuffling and other mechanisms during genome evolution (Keren et al. 2010), so we checked both exons for congruence. Even though humans and birds are separated by hundreds of millions of years of evolution (~ 300 mya), both have retained very similar exon structure; this level of conservation also extends to both exon 6 and exon 12 (e.g., exon 12 humans = exon 12 in ducks; Supp. Fig. 3). Thus, it is appropriate to extrapolate results from what is known about similar variation in the same exons of other organisms, and how it might relate back our outlier variants in the Andean duck species.

Specifically, variation in exon 12 in humans has been implicated with both loss-of-function and gain-of-function mutations associated with RBC production. Such gain-of-function mutations are known to cause erythrocytosis, resulting in an increased number of RBCs through its regulation of erythropoietan, in combination with pulmonary arterial hypertension, in humans (Gale et al. 2008; Percy et al. 2008a; Percy et al. 2008b). Cattle housed at high-altitude also have similar gain-of-function variants in exon 12 associated with pulmonary hypertension (Newman et al. 2015). Although, in both the human and cattle studies, those variants were largely considered deleterious, they demonstrate the ability for genetic variation in exon 12 of *EPAS1* to have direct physiological effects similar to those frequently targeted by selection in hypoxic environments. In addition, our results showed that speckled teal had additional outliers in *EPAS1* located in exon 6, which has been identified in human high-altitude populations to be responsible for adaptive changes in heart rate and hypertension (Buroker et al. 2012; Yi et al. 2010). Our results mirror variation in *EPAS1* in other human populations, in the sense that the gene itself is a target of selection; however, high-altitude adaptation in Tibetan and Andean populations have only shown evidence for variation in non-protein-coding regions of *EPAS1*, unlike our results that show significant variants in both protein-coding and non-protein-coding regions of *EPAS1*.

Exactly which mechanistic avenues these Andean duck species are using is unclear, given the current data; yet, it is interesting to note that the speckled teal, like Tibetan humans, are characterized by Hb levels more similar that of their low-altitude counterparts, unlike other some Andean duck populations, like yellow-billed pintail and cinnamon teal (McCracken, unpublished data), who show elevated Hb like Andean people (Beall 2007; Lague et al. 2017). This could suggest a connection between *EPAS1*, *EGLN1*, and Hb production, especially based on what we know about Tibetan and Andean populations whose genetic variation in both genes has been implicated in protecting individuals from erythrocytosis/polycythemia, resulting from living at high-altitude (Peng et al. 2017; Peng et al. 2011; Wu and Kayser 2006). With potentially causative exonic variation present in *EPAS1* and intronic variation in *EGLN1*, the relationship between these two genes and potential avenues for high-altitude adaptation is similar to those found in human populations. The ultimate hypothesis from our results is that a combination of function-altering (either gain- or loss-of function) mutations in *EPAS1*, and transcriptional regulation differences between *EGLN1* haplotype variants, may be causing changes in downstream target transactivation, resulting in a blunted hypoxic response. Testing this connection between these variants and such a physiological response would require future functional assessment (next section).

### Future functional assessment of EGLN1 and EPAS1 in Andean waterfowl

Despite similar results from other species, it is difficult to say with certainty what effect these variants actually have in the waterfowl species assayed in this study, without functional assessment at the physiological level. The future goal would be to assess these variants in a more causal way than the outlier analyses presented in this study; however, there is some difficulty in performing direct assessment due to the issue of *EPAS1* not having a full protein structure available (i.e., lacking the ODD/NTAD/CTAD domains), in addition to the general issues of performing functional genomics in a wild vertebrate species, with no captive caught populations.

It is important to note that, as it relates to functional assessment of variants for either *EPAS1* or *EGLN1* found in the more prominent human studies, currently none have been directly assessed (i.e., site-directed mutagenesis); instead, they have been indirectly assessed through whole-scale gene knockdowns in mice and tissue culture, and/or measurements of various physiological differences (Peng et al. 2017; Xiang et al. 2013). Even though our extrapolation from previous functional work on the same genes is somewhat circumstantial in respect to the organisms used in this study, we feel that these results from other organisms will potentially help guide future experiments concerning how best to assay the role of the variants in enabling high-altitude living found in this study.

## Conclusions

Hypoxia as a stressor in high-altitude environments has been shown to have facilitated a great degree of convergence across many animals, but especially in the case of high-altitude human and Andean duck populations, as demonstrated by the results of this research. Ultimately, we were able to show a potentially high degree of convergence between high-altitude human and duck populations on the HIF pathway itself, but also that convergence included two gene members, *EPAS1* and *EGLN1*. In addition, between the high-altitude populations of speckled teal and yellow-billed pintail, nonsynonymous changes in specific exons (exon 12) in close proximity to protein domains associated with O_2_-driven protein stability and transactivation suggests evidence of strong molecular convergence. Although the specific molecular mechanisms associated with these variants are currently unknown in this system, previous work from other organisms suggests that these variants are likely resulting in a blunted hypoxic response, potentially through expression of Hb and other downstream targets. Ultimately, we were able to identify a potential molecular mechanism for high-altitude adaptation in Andean duck species, through the HIF pathway; in addition, the results also highlight how frequently natural selection can select for the same genes and pathways in response to a similar selective pressure, resulting in convergent mechanisms of adaptation to similar environments.

### Data archiving

Parsed Illumina reads for the RAD-seq data sets are deposited in the NCBI short read archive (SRA PRJEB11624). Raw Illumina reads for the target-enrichment data sets are deposited in the NCBI short reach archive (SRA PRJNA508951). A Dryad digital repository houses additional data files (10.5061/dryad.kd08516). Scripts and other small files associated with analysis are available on GitHub (https://github.com/amgraham07).

## Supplementary information

SUPP Figure 1

SUPP Figure 3

SUPP Figure 2

SUPP Table 1

SUPP Table 2

SUPP Table 3

SUPP Table 4

SUPP Table 5

Supplemental figure titels
