# Supplementary figures and images for: Convergent evolution on the hypoxia-inducible factor (HIF) pathway genes EGLN1 and EPAS1 in high-altitude ducks
Source: Heredity (Edinb). 2019 Jan 10;122(6):819–32. doi: 10.1038/s41437-018-0173-z (PMC6781116; doi:10.1038/s41437-018-0173-z)

# HIF-1 SIGNALING PATHWAY

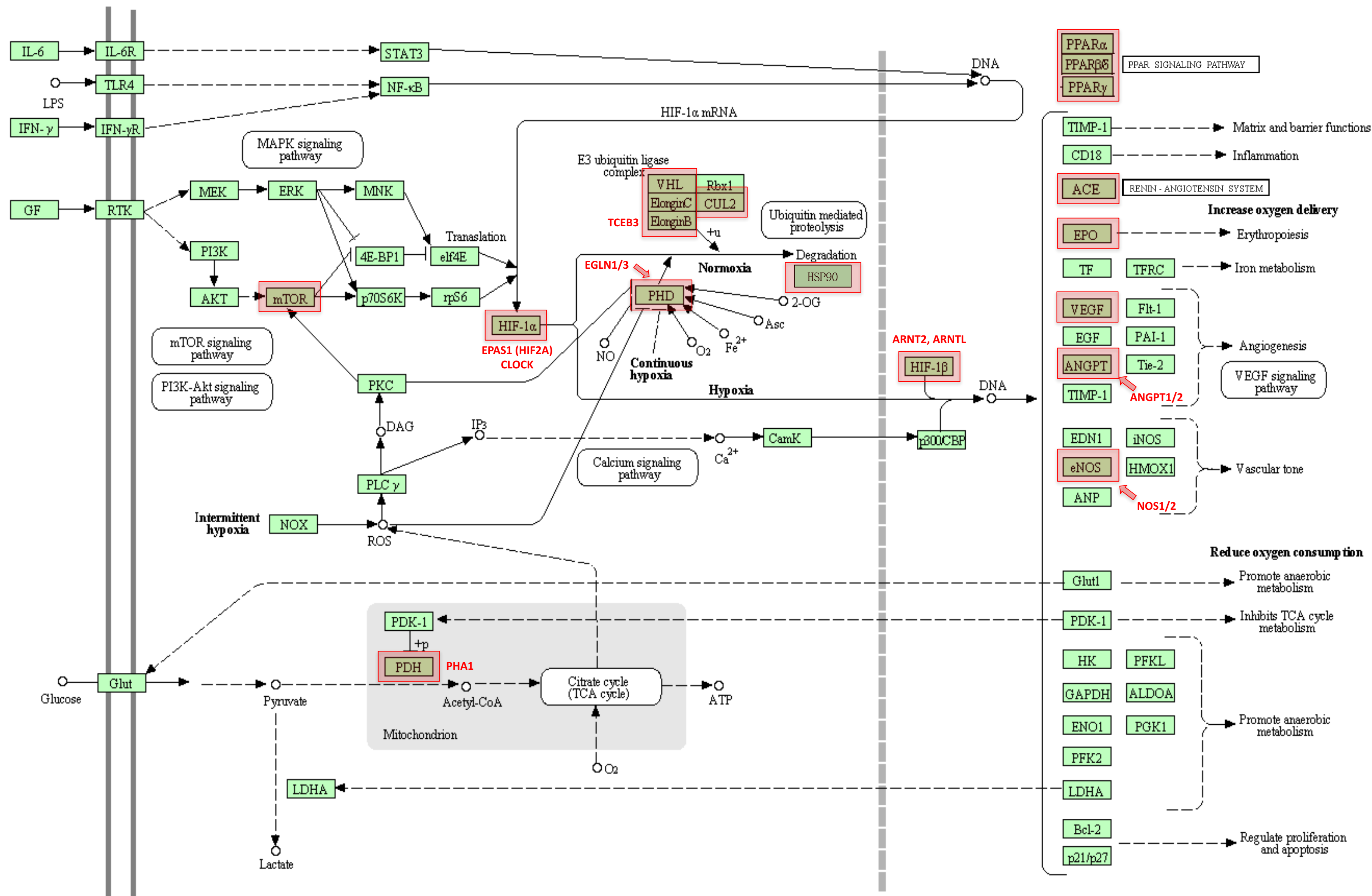

Supplement: Supplementary file 1 — SUPP Figure 1 [file 41437_2018_173_MOESM1_ESM.pdf]

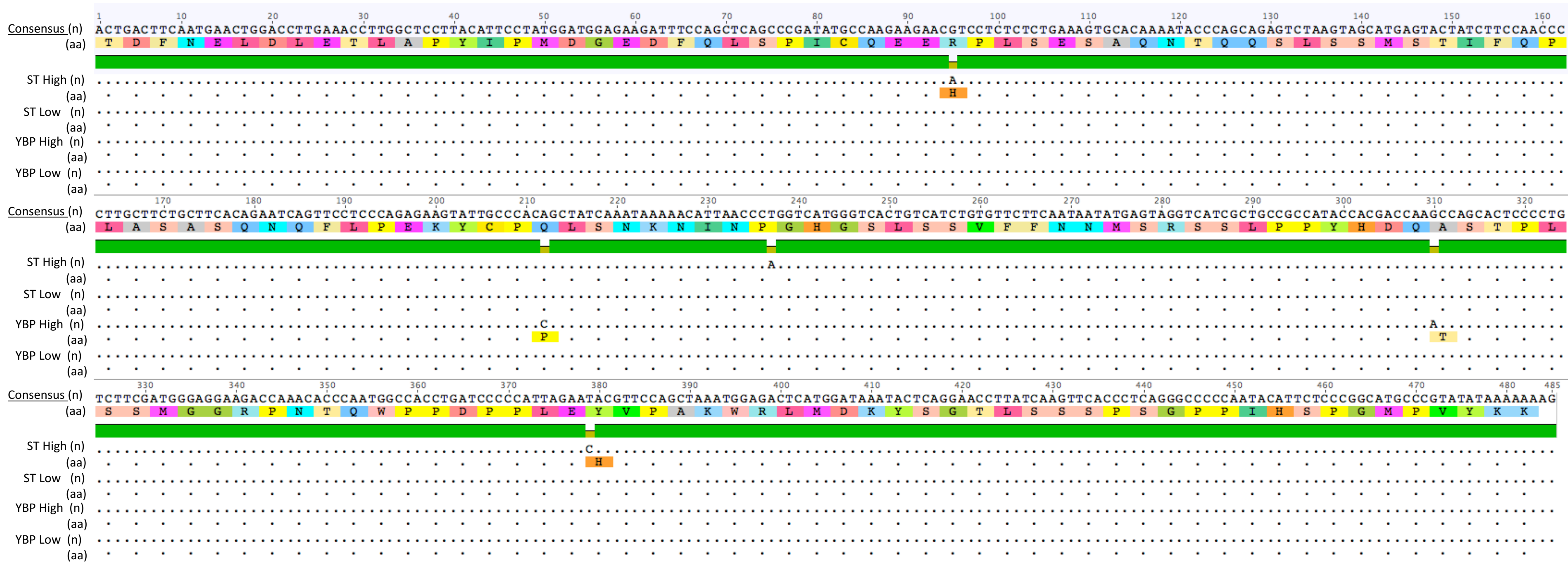

Supplement: Supplementary file 3 — SUPP Figure 2 [file 41437_2018_173_MOESM3_ESM.pdf]
