# Supplementary material for: Convergent evolution on the hypoxia-inducible factor (HIF) pathway genes EGLN1 and EPAS1 in high-altitude ducks
Source: Heredity (Edinb). 2019 Jan 10;122(6):819–32. doi: 10.1038/s41437-018-0173-z (PMC6781116; doi:10.1038/s41437-018-0173-z)
Supplement: Supplementary file 2 — SUPP Figure 3 [file 41437_2018_173_MOESM2_ESM.pdf]

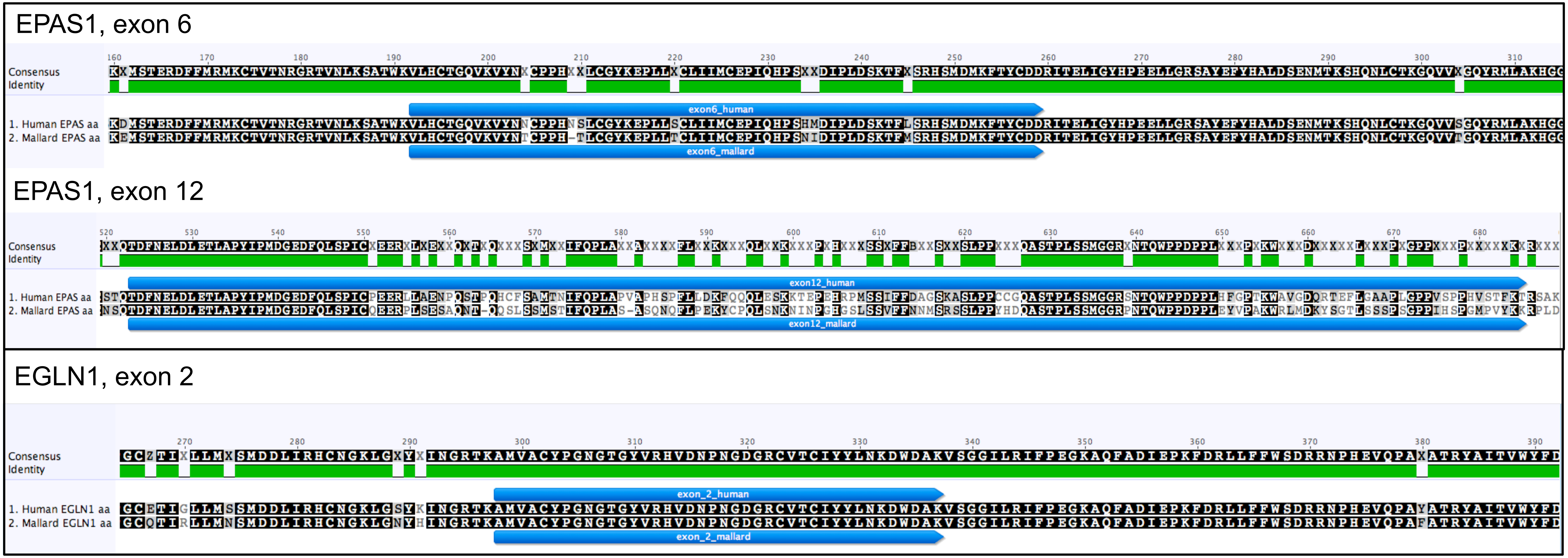
EPAS1, exon 6
Consensus
Identity
exon6_human
1. Human EPAS aa
2. Mallard EPAS aa
exon6_mallard
EPAS1, exon 12
Consensus
Identity
exon12_human
1. Human EPAS aa
2. Mallard EPAS aa
exon12_mallard
EGLN1, exon 2
Consensus
Identity
exon_2_human
1. Human EGLN1 aa
2. Mallard EGLN1 aa
exon_2_mallard
