# Supplementary material for: Convergent evolution on the hypoxia-inducible factor (HIF) pathway genes EGLN1 and EPAS1 in high-altitude ducks
Source: Heredity (Edinb). 2019 Jan 10;122(6):819–32. doi: 10.1038/s41437-018-0173-z (PMC6781116; doi:10.1038/s41437-018-0173-z)
Supplement: Supplementary file 4 — SUPP Table 1 [file 41437_2018_173_MOESM4_ESM.pdf]

SUPP Table 1: List of HIF signaling pathway members, their location in the mallard genome (ENSEMBL), including the number of probes/baits designed for each gene (MYcroarray).

Gene Name	Location in Duck Genome (ENSEMBL)	begin	end	total length	# Probes
ACE	Scaffold KB744725.1: 386,729-403,257	386729	403257	16528	23
ACE2	Scaffold KB742701.1: 1,766,330-1,789,804	1766330	1789804	23474	-
ANGPT1	Scaffold KB743875.1: 238,671-419,023	238671	419023	180352	447
ANGPT2	Scaffold KB742406.1: 1,290,414-1,334,615	1290414	1334615	44201	78
ARNT	Scaffold KB744049.1: 20,467-38,811	20467	38811	18344	13
ARNT2	Scaffold KB744113.1: 278,897-358,152	278897	358152	79255	81
ARNTL	Scaffold KB744206.1: 57,975-84,046	57975	84046	26071	30
CLOCK	Scaffold KB742619.1: 827,048-854,490	827048	854490	27442	29
CUL2	Scaffold KB742539.1: 2,267,987-2,311,340	2267987	2311340	43353	36
EDN1	Scaffold KB743575.1: 623,460-625,674	623460	625674	2214	-
EGLN1	Scaffold KB743594.1: 39,865-71,284	39865	71284	31419	52
EGLN3	Scaffold KB743383.1: 66,183-90,875	66183	90875	24692	25
EPAS1/HIF2a	Scaffold KB742444.1: 734,232-772,478	734232	772478	38246	64
FIH/HIF1AN	Scaffold KB743728.1: 852,333-858,443	852333	858443	6110	-
HIF1A	Scaffold KB745078.1: 277,272-293,388	277272	293388	16116	7
HSP90AA1	Scaffold KB743197.1: 687,101-694,368	687101	694368	7267	7
MTOR	Scaffold KB743246.1: 258,381-322,791	258381	322791	64410	45
NOS1	Scaffold KB744108.1: 141,687-174,300	141687	174300	32613	4
NOS2	Scaffold KB743217.1: 377,684-396,078	377684	396078	18394	13
P4HA1	Scaffold KB744430.1: 583,757-609,851	583757	609851	26094	31
P4HA2	Scaffold KB743591.1: 85,973-106,217	85973	106217	20244	11
P4HA3	Scaffold KB744216.1: 262,001-267,897	262001	267897	5896	5
PDHA1	Scaffold KB742701.1: 132,835-143,350	132835	143350	10515	16
PDHB	Scaffold KB744074.1: 915,372-919,000	915372	919000	3628	-
PPARA (alpha)	Scaffold KB742459.1: 937,145-969,765	937145	969765	32620	47
PPARD (delta)	Scaffold KB743510.1: 216,148-220,291	216148	220291	4143	2
PPARG (gamma)	Scaffold KB742679.1: 37,634-61,994	37634	61994	24360	34
TCEB3	Scaffold KB742637.1: 387,311-394,427	387311	394427	7116	3
THPO	Scaffold KB743359.1: 685,580-686,504	685580	686504	924	-
THRB	Scaffold KB744720.1: 282,100-308,664	282100	308664	26564	44
VEGF	Scaffold KB742809.1: 508,129-521,531	508129	521531	13402	34
