# Supplementary material for: Convergent evolution on the hypoxia-inducible factor (HIF) pathway genes EGLN1 and EPAS1 in high-altitude ducks
Source: Heredity (Edinb). 2019 Jan 10;122(6):819–32. doi: 10.1038/s41437-018-0173-z (PMC6781116; doi:10.1038/s41437-018-0173-z)
Supplement: Supplementary file 5 — SUPP Table 2 [file 41437_2018_173_MOESM5_ESM.pdf]

<u>SUPP Table 2</u>: Assembly statistics for the 26 genes sequenced through target-enrichment – "depth of coverage" represents the number of reads that included in the reconstructed sequence against the mallard reference; "gene coveraged" refers to the total amount of the mallard reference that included any amount of coverage.

	Speckled teal		***Yellow-billed pintail***		***Cinnamon teal***		***All Three***	
Gene Name	***Depth of Coverage***	***Gene Covered***	***Depth of Coverage***	***Gene Covered***	***Depth of Coverage***	***Gene Covered***	***Depth of Coverage***	***Gene Covered***
ACE	147	97.70%	157	99.20%	165	99.20%	156.33	98.70%
ANGPT1	456	95.50%	463	96.10%	492	94.60%	470.33	95.40%
ANGPT2	514	99.40%	518	99.60%	570	99.60%	534.00	99.53%
ARNT	411	99.50%	428	99.80%	437	99.80%	425.33	99.70%
ARNT2	463	99.80%	478	99.90%	520	99.90%	487.00	99.87%
ARNTL	558	91.70%	567	92.10%	601	90.90%	575.33	91.57%
CLOCK	463	99.80%	471	100.00%	497	100.00%	477.00	99.93%
CUL2	589	99.90%	592	100.00%	572	98.00%	584.33	99.30%
EGLN1	388	99.30%	404	99.70%	398	99.60%	396.67	99.53%
EGLN3	421	99.30%	436	99.60%	460	99.70%	439.00	99.53%
HIF2A	456	99.70%	457	100.00%	485	100.00%	466.00	99.90%
HIF1A	512	99.90%	523	100.00%	546	100.00%	527.00	99.97%
HSP90AA1	476	99.90%	474	100.00%	405	100.00%	451.67	99.97%
MTOR	476	99.80%	485	100.00%	510	100.00%	490.33	99.93%
NOS1	140	95.20%	151	96.60%	152	96.10%	147.67	95.97%
NOS2	540	100.00%	549	100.00%	602	99.90%	563.67	99.97%
P4HA1	406	98.00%	413	98.50%	455	98.40%	424.67	98.30%
P4HA2	509	99.70%	514	99.90%	569	99.80%	530.67	99.80%
P4HA3	72	79.80%	78	81.80%	72	81.50%	74.00	81.03%
PDHA1	507	100.00%	506	99.90%	500	99.60%	504.33	99.83%
PPARA	532	98.40%	557	98.60%	596	97.70%	561.67	98.23%
PPARD	119	98.20%	132	100.00%	121	99.30%	124.00	99.17%
PPARG	516	99.70%	523	99.70%	565	99.80%	534.67	99.73%
TCEB3	127	95.70%	143	97.80%	128	96.60%	132.67	96.70%
THRB	430	99.50%	439	99.90%	492	99.80%	453.67	99.73%
VEGF	219	99.00%	229	99.80%	234	99.80%	227.33	99.53%
