# Supplementary material for: Convergent evolution on the hypoxia-inducible factor (HIF) pathway genes EGLN1 and EPAS1 in high-altitude ducks
Source: Heredity (Edinb). 2019 Jan 10;122(6):819–32. doi: 10.1038/s41437-018-0173-z (PMC6781116; doi:10.1038/s41437-018-0173-z)
Supplement: Supplementary file 6 — SUPP Table 3 [file 41437_2018_173_MOESM6_ESM.pdf]

SUPP Table 3: Outlier list from MCHEZA in cinnamon teal, including the SNP variants associated with the HIF-pathway (and corresponding position on the gene) who met the FDR > 0.99, and who also were in the top 1% of F_{ST} values; Het = heterozygosity.

#CHROM	POS	Locus	Het	Fst	P(Simul Fst<sample Fst)
**NOS1_KB744108.1**	23772	SNP_20649	0.515465	0.823984	1
**NOS1_KB744108.1**	23724	SNP_20644	0.496403	0.799555	1
**NOS1_KB744108.1**	23747	SNP_20647	0.496403	0.799555	1
**NOS1_KB744108.1**	23835	SNP_20654	0.496403	0.799555	1
**NOS1_KB744108.1**	23680	SNP_20642	0.45139	0.757868	1
**EPAS1_KB742444.1**	12059	SNP_14947	0.483233	0.753212	1
**EPAS1_KB742444.1**	14303	SNP_15034	0.483233	0.753212	1
**EPAS1_KB742444.1**	14502	SNP_15044	0.483233	0.753212	1
**NOS1_KB744108.1**	20356	SNP_20439	0.45071	0.727606	1
**EPAS1_KB742444.1**	9066	SNP_14804	0.491823	0.718793	1
**EPAS1_KB742444.1**	9111	SNP_14808	0.491823	0.718793	1
**EPAS1_KB742444.1**	9432	SNP_14823	0.491823	0.718793	1
**EPAS1_KB742444.1**	9873	SNP_14842	0.491823	0.718793	1
**EPAS1_KB742444.1**	12027	SNP_14944	0.491823	0.718793	1
**EPAS1_KB742444.1**	12779	SNP_14978	0.491823	0.718793	1
**EPAS1_KB742444.1**	12964	SNP_14984	0.491823	0.718793	1
**EPAS1_KB742444.1**	14672	SNP_15051	0.498905	0.683147	1
**NOS1_KB744108.1**	9350	SNP_19742	0.266267	0.679482	1
**PPARA_KB742459.1**	4399	SNP_24908	0.345688	0.663169	1
**EPAS1_KB742444.1**	20362	SNP_15278	0.504302	0.646132	1
**CLOCK_KB742619.1**	17325	SNP_10768	0.255142	0.614556	1
**EPAS1_KB742444.1**	24950	SNP_15428	0.512892	0.610632	1
**EPAS1_KB742444.1**	14414	SNP_15040	0.507798	0.607577	1
**EPAS1_KB742444.1**	21693	SNP_15329	0.507798	0.607577	1
**EPAS1_KB742444.1**	24204	SNP_15398	0.462104	0.599203	1
**EPAS1_KB742444.1**	24566	SNP_15418	0.462104	0.599203	1
**EGLN1_KB743594.1**	5005	SNP_12867	0.509129	0.567272	1
**EPAS1_KB742444.1**	15292	SNP_15068	0.509129	0.567272	1
**EPAS1_KB742444.1**	25792	SNP_15458	0.509129	0.567272	1
**EPAS1_KB742444.1**	26691	SNP_15504	0.509129	0.567272	1
**NOS1_KB744108.1**	19393	SNP_20386	0.509129	0.567272	1
**NOS1_KB744108.1**	19394	SNP_20387	0.509129	0.567272	1
**CLOCK_KB742619.1**	17366	SNP_10773	0.28188	0.564621	1
**EGLN1_KB743594.1**	29894	SNP_13921	0.486069	0.535239	1
**EPAS1_KB742444.1**	15502	SNP_15077	0.356756	0.526242	1
**EGLN1_KB743594.1**	24029	SNP_13659	0.507958	0.524956	1
**EGLN1_KB743594.1**	24100	SNP_13664	0.507958	0.524956	1
**EPAS1_KB742444.1**	12684	SNP_14972	0.507958	0.524956	1
**EPAS1_KB742444.1**	12698	SNP_14973	0.507958	0.524956	1
**EPAS1_KB742444.1**	13046	SNP_14991	0.507958	0.524956	1
**EPAS1_KB742444.1**	13785	SNP_15012	0.507958	0.524956	1

**EPAS1_KB742444.1**	15422	SNP_15074	0.507958	0.524956	1
**EPAS1_KB742444.1**	15887	SNP_15091	0.507958	0.524956	1
**EPAS1_KB742444.1**	17001	SNP_15156	0.507958	0.524956	1
**EPAS1_KB742444.1**	17083	SNP_15160	0.507958	0.524956	1
**EPAS1_KB742444.1**	20799	SNP_15297	0.507958	0.524956	1
**EPAS1_KB742444.1**	23022	SNP_15365	0.507958	0.524956	1
**MTOR_KB743246.1**	29375	SNP_17813	0.507958	0.524956	1
**NOS1_KB744108.1**	24359	SNP_20684	0.507958	0.524956	1
**P4HA3_KB744216.1**	3770	SNP_23934	0.507958	0.524956	1
**EPAS1_KB742444.1**	3914	SNP_14659	0.483233	0.518324	1
**EPAS1_KB742444.1**	4012	SNP_14663	0.483233	0.518324	1
**EPAS1_KB742444.1**	4018	SNP_14664	0.483233	0.518324	1
**EPAS1_KB742444.1**	4038	SNP_14666	0.483233	0.518324	1
**EPAS1_KB742444.1**	24169	SNP_15397	0.483233	0.518324	1
