# Supplementary material for: Convergent evolution on the hypoxia-inducible factor (HIF) pathway genes EGLN1 and EPAS1 in high-altitude ducks
Source: Heredity (Edinb). 2019 Jan 10;122(6):819–32. doi: 10.1038/s41437-018-0173-z (PMC6781116; doi:10.1038/s41437-018-0173-z)
Supplement: Supplementary file 7 — SUPP Table 4 [file 41437_2018_173_MOESM7_ESM.pdf]

SUPP Table 4: Outlier list in yellow-billed pintail, including the SNP variants associated with the HIF-pathway (and corresponding position on the gene) who [1] were in the top 1% of of F_{ST} values, [2] met the FDR > 0.99 in MCHEZA, and [3] whose Log10(PO) > 0.5 (ie."substantial") in BayeScan.

MCHEZA + 99th percentile (>0.710)						BayeScan (Log10(PO)>0.5 = "substantial")								
#CHROM	POS	Locus_MC	Het	Fst	P(Simul Fst<sample Fst)	#CHROM	POS	Pos_BS	prob	log10(PO)	qval	alpha	Simul Fst	Exon Overlap
EGLN1_KB743594.1	881	SNP_13409	0.462104	0.818823	1	EGLN1_KB743594.1	881	13409	0.9956	2.3545	0.0028542	2.0586	0.32283	-
EGLN1_KB743594.1	2779	SNP_13483	0.473285	0.786521	1	EGLN1_KB743594.1	2779	13483	0.96439	1.4327	0.008721	1.9034	0.29609	-
EGLN1_KB743594.1	2914	SNP_13489	0.473285	0.786521	1	EGLN1_KB743594.1	2914	13489	0.96619	1.4561	0.0053532	1.917	0.29872	-
EGLN1_KB743594.1	3559	SNP_13529	0.483233	0.753212	1	EGLN1_KB743594.1	3559	13529	0.83417	0.70158	0.035227	1.601	0.2541	-
EGLN1_KB743594.1	5997	SNP_13653	0.462104	0.818823	1	EGLN1_KB743594.1	5997	13653	0.998	2.698	0.0016003	2.0389	0.31886	Exon2
EGLN1_KB743594.1	9377	SNP_13782	0.483233	0.753212	1	EGLN1_KB743594.1	9377	13782	0.83357	0.6997	0.036653	1.5882	0.25194	-
EGLN1_KB743594.1	27855	SNP_14401	0.473285	0.786521	1	EGLN1_KB743594.1	27855	14401	0.90678	0.988	0.023966	1.7225	0.26854	-
EPAS1_KB742444.1	2599	SNP_15167	0.422162	0.71501	1	EPAS1_KB742444.1	2599	15167	0.9974	2.5838	0.0021583	1.9724	0.30429	-
EPAS1_KB742444.1	2621	SNP_15168	0.422162	0.71501	1	EPAS1_KB742444.1	2621	15168	0.9978	2.6565	0.0018004	1.9712	0.30403	-
EPAS1_KB742444.1	2631	SNP_15169	0.422162	0.71501	1	EPAS1_KB742444.1	2631	15169	0.9972	2.5515	0.0023125	1.9562	0.30137	-
EPAS1_KB742444.1	6305	SNP_15275	0.422162	0.71501	1	EPAS1_KB742444.1	6305	15275	0.9966	2.467	0.0024779	1.9689	0.3039	-
EPAS1_KB742444.1	25376	SNP_16045	0.422162	0.71501	1	EPAS1_KB742444.1	25376	16045	0.9962	2.4185	0.0026177	1.967	0.30361	-
EPAS1_KB742444.1	26021	SNP_16068	0.422162	0.71501	1	EPAS1_KB742444.1	26021	16068	0.997	2.5215	0.0023862	1.97	0.30383	-
EPAS1_KB742444.1	26519	SNP_16089	0.422162	0.71501	1	EPAS1_KB742444.1	26519	16089	0.9984	2.7951	0.0010002	1.9638	0.30268	-
EPAS1_KB742444.1	27470	SNP_16110	0.422162	0.71501	1	EPAS1_KB742444.1	27470	16110	0.9958	2.3748	0.0027749	1.9537	0.30089	-
EPAS1_KB742444.1	27546	SNP_16115	0.422162	0.71501	1	EPAS1_KB742444.1	27546	16115	0.9976	2.6187	0.0020404	1.9745	0.30523	-
EPAS1_KB742444.1	27862	SNP_16126	0.483233	0.753212	1	EPAS1_KB742444.1	27862	16126	0.83637	0.70853	0.030786	1.6061	0.25489	-
EPAS1_KB742444.1	28384	SNP_16160	0.422162	0.71501	1	EPAS1_KB742444.1	28384	16160	0.9968	2.4934	0.0024143	1.97	0.30406	-
EPAS1_KB742444.1	28474	SNP_16166	0.422162	0.71501	1	EPAS1_KB742444.1	28474	16166	0.9966	2.467	0.0024779	1.9787	0.30543	-
EPAS1_KB742444.1	28583	SNP_16174	0.422162	0.71501	1	EPAS1_KB742444.1	28583	16174	0.9972	2.5515	0.0023125	1.9497	0.30017	-
EPAS1_KB742444.1	29121	SNP_16199	0.422162	0.71501	1	EPAS1_KB742444.1	29121	16199	0.9976	2.6187	0.0020404	1.9659	0.30307	-
EPAS1_KB742444.1	30636	SNP_16238	0.422162	0.71501	1	EPAS1_KB742444.1	30636	16238	0.9962	2.4185	0.0026177	1.9692	0.30389	-
EPAS1_KB742444.1	30642	SNP_16239	0.422162	0.71501	1	EPAS1_KB742444.1	30642	16239	0.998	2.698	0.0016003	1.9788	0.3058	-
EPAS1_KB742444.1	30855	SNP_16257	0.422162	0.71501	1	EPAS1_KB742444.1	30855	16257	0.9956	2.3545	0.0028542	1.9578	0.30169	-
EPAS1_KB742444.1	30872	SNP_16258	0.422162	0.71501	1	EPAS1_KB742444.1	30872	16258	0.9978	2.6565	0.0018004	1.9581	0.30135	-
EPAS1_KB742444.1	31342	SNP_16265	0.422162	0.71501	1	EPAS1_KB742444.1	31342	16265	0.9972	2.5515	0.0023125	1.9658	0.30341	-

EPAS1_KB742444.1	31362	SNP_16267	0.422162	0.71501	1	EPAS1_KB742444.1	31362	16267	0.9976	2.6187	0.0020404	1.9625	0.30187	-
EPAS1_KB742444.1	31413	SNP_16273	0.422162	0.71501	1	EPAS1_KB742444.1	31413	16273	0.9974	2.5838	0.0021583	1.9683	0.30328	-
EPAS1_KB742444.1	31491	SNP_16278	0.422162	0.71501	1	EPAS1_KB742444.1	31491	16278	0.997	2.5215	0.0023862	1.9641	0.30264	-
EPAS1_KB742444.1	31559	SNP_16280	0.422162	0.71501	1	EPAS1_KB742444.1	31559	16280	0.9972	2.5515	0.0023125	1.9612	0.30191	-
EPAS1_KB742444.1	32499	SNP_16314	0.422162	0.71501	1	EPAS1_KB742444.1	32499	16314	0.9974	2.5838	0.0021583	1.9655	0.30336	-
EPAS1_KB742444.1	32739	SNP_16318	0.422162	0.71501	1	EPAS1_KB742444.1	32739	16318	0.9972	2.5515	0.0023125	1.9528	0.30062	Exon 12
EPAS1_KB742444.1	32837	SNP_16324	0.422162	0.71501	1	EPAS1_KB742444.1	32837	16324	0.9972	2.5515	0.0023125	1.9552	0.30085	Exon 12
EPAS1_KB742444.1	33197	SNP_16331	0.422162	0.71501	1	EPAS1_KB742444.1	33197	16331	0.9976	2.6187	0.0020404	1.9575	0.30142	-
EPAS1_KB742444.1	33971	SNP_16360	0.422162	0.71501	1	EPAS1_KB742444.1	33971	16360	0.997	2.5215	0.0023862	1.9591	0.30137	-
EPAS1_KB742444.1	34510	SNP_16367	0.422162	0.71501	1	EPAS1_KB742444.1	34510	16367	0.996	2.3961	0.0026561	1.987	0.30728	-
EPAS1_KB742444.1	34514	SNP_16368	0.422162	0.71501	1	EPAS1_KB742444.1	34514	16368	0.9982	2.7439	0.0014003	1.9677	0.30342	-
EPAS1_KB742444.1	34516	SNP_16369	0.422162	0.71501	1	EPAS1_KB742444.1	34516	16369	0.9964	2.442	0.002546	1.9738	0.30496	-
EPAS1_KB742444.1	34519	SNP_16370	0.422162	0.71501	1	EPAS1_KB742444.1	34519	16370	0.9978	2.6565	0.0018004	1.9739	0.30495	-
EPAS1_KB742444.1	34619	SNP_16373	0.422162	0.71501	1	EPAS1_KB742444.1	34619	16373	0.9976	2.6187	0.0020404	1.9642	0.30255	-
EPAS1_KB742444.1	34677	SNP_16376	0.422162	0.71501	1	EPAS1_KB742444.1	34677	16376	0.9974	2.5838	0.0021583	1.9639	0.30276	-
