# Supplementary material for: Convergent evolution on the hypoxia-inducible factor (HIF) pathway genes EGLN1 and EPAS1 in high-altitude ducks
Source: Heredity (Edinb). 2019 Jan 10;122(6):819–32. doi: 10.1038/s41437-018-0173-z (PMC6781116; doi:10.1038/s41437-018-0173-z)
Supplement: Supplementary file 8 — SUPP Table 5 [file 41437_2018_173_MOESM8_ESM.pdf]

SUPP Table 5: Outlier list in speckled teal, including the SNP variants associated with the HIF-pathway (and corresponding position on the gene) who [1] were in the top 1% of F_{ST} values, [2] met the FDR > 0.99 in MCHEZA, and [3] whose Log10(PO) > 0.5 (ie."substantial") in BayeScan.

MCHEZA + 99th percentil Fst (>0.889)						***BayeScan (Log10(PO)>0.5 = "substantial")***									
#CHROM	***POS***	***Locus_MC***	***Het***	***Fst***	***P(Simul Fst<sample Fst)***	***#CHROM***	***POS***	***Pos_BS***	***prob***	***log10(PO)***	***qval***	***alpha***	***Simul Fst***	***Exon Overlap***
EGLN1_KB743594.1	16425	SNP_12283	0.422162	0.910485	1	EGLN1_KB743594.1	16425	12283	0.86977	0.82471	0.060736	1.491	0.51713	-
EGLN1_KB743594.1	16427	SNP_12284	0.422162	0.910485	1	EGLN1_KB743594.1	16427	12284	0.94759	1.2572	0.050477	1.7731	0.57711	-
EPAS1_KB742444.1	13775	SNP_14052	0.422162	0.910485	1	EPAS1_KB742444.1	13775	14052	0.93439	1.1535	0.05595	1.7743	0.57747	-
EPAS1_KB742444.1	14848	SNP_14085	0.422162	0.910485	1	EPAS1_KB742444.1	14848	14085	0.94779	1.259	0.04951	1.7806	0.57862	Exon 6
EPAS1_KB742444.1	15026	SNP_14089	0.422162	0.910485	1	EPAS1_KB742444.1	15026	14089	0.95379	1.3147	0.046209	1.7915	0.58056	-
EPAS1_KB742444.1	16831	SNP_14193	0.422162	0.910485	1	EPAS1_KB742444.1	16831	14193	0.94319	1.2202	0.053068	1.7815	0.5786	-
EPAS1_KB742444.1	16832	SNP_14194	0.422162	0.910485	1	EPAS1_KB742444.1	16832	14194	0.94299	1.2185	0.053331	1.7792	0.57807	-
EPAS1_KB742444.1	26494	SNP_14522	0.422162	0.910485	1	EPAS1_KB742444.1	26494	14522	0.94279	1.2169	0.053573	1.77	0.5762	-
EPAS1_KB742444.1	28232	SNP_14581	0.422162	0.910485	1	EPAS1_KB742444.1	28232	14581	0.94419	1.2283	0.052511	1.7804	0.57853	-
EPAS1_KB742444.1	29126	SNP_14616	0.422162	0.910485	1	EPAS1_KB742444.1	29126	14616	0.94399	1.2267	0.05278	1.7757	0.57733	-
EPAS1_KB742444.1	29716	SNP_14637	0.422162	0.910485	1	EPAS1_KB742444.1	29716	14637	0.94099	1.2026	0.054521	1.7959	0.58165	-
EPAS1_KB742444.1	30099	SNP_14640	0.422162	0.910485	1	EPAS1_KB742444.1	30099	14640	0.94219	1.2121	0.054044	1.7845	0.57909	-
EPAS1_KB742444.1	30102	SNP_14641	0.422162	0.910485	1	EPAS1_KB742444.1	30102	14641	0.94219	1.2121	0.054044	1.784	0.57919	-
EPAS1_KB742444.1	30332	SNP_14647	0.422162	0.910485	1	EPAS1_KB742444.1	30332	14647	0.94499	1.235	0.05221	1.7813	0.57881	-
EPAS1_KB742444.1	31922	SNP_14702	0.423029	0.904802	1	EPAS1_KB742444.1	31922	14702	0.89318	0.92228	0.058255	1.6688	0.5548	-
EPAS1_KB742444.1	32283	SNP_14719	0.437598	0.909661	1	EPAS1_KB742444.1	32283	14719	0.93039	1.126	0.056456	1.7548	0.57298	-
EPAS1_KB742444.1	32384	SNP_14722	0.422162	0.910485	1	EPAS1_KB742444.1	32384	14722	0.94139	1.2058	0.054285	1.7719	0.57699	-
EPAS1_KB742444.1	32388	SNP_14723	0.422162	0.910485	1	EPAS1_KB742444.1	32388	14723	0.93979	1.1933	0.055563	1.7786	0.57794	-
EPAS1_KB742444.1	32410	SNP_14725	0.422162	0.910485	1	EPAS1_KB742444.1	32410	14725	0.94019	1.1964	0.055369	1.7745	0.57717	-
EPAS1_KB742444.1	32411	SNP_14726	0.422162	0.910485	1	EPAS1_KB742444.1	32411	14726	0.94539	1.2383	0.05193	1.7724	0.57668	-
EPAS1_KB742444.1	32412	SNP_14727	0.422162	0.910485	1	EPAS1_KB742444.1	32412	14727	0.94059	1.1995	0.054754	1.7586	0.57385	-
EPAS1_KB742444.1	32449	SNP_14729	0.422162	0.910485	1	EPAS1_KB742444.1	32449	14729	0.94039	1.198	0.055176	1.7724	0.57662	-
EPAS1_KB742444.1	32453	SNP_14730	0.422162	0.910485	1	EPAS1_KB742444.1	32453	14730	0.94739	1.2555	0.050782	1.7792	0.57848	-
EPAS1_KB742444.1	32497	SNP_14733	0.422162	0.910485	1	EPAS1_KB742444.1	32497	14733	0.94539	1.2383	0.05193	1.7725	0.57647	-
EPAS1_KB742444.1	32501	SNP_14734	0.422162	0.910485	1	EPAS1_KB742444.1	32501	14734	0.94899	1.2696	0.04861	1.7892	0.58042	-
EPAS1_KB742444.1	32622	SNP_14735	0.422162	0.910485	1	EPAS1_KB742444.1	32622	14735	0.94539	1.2383	0.05193	1.7877	0.57993	Exon 12
EPAS1_KB742444.1	32764	SNP_14739	0.422162	0.910485	1	EPAS1_KB742444.1	32764	14739	0.94759	1.2572	0.050477	1.7875	0.5804	Exon 12
EPAS1_KB742444.1	32906	SNP_14745	0.422162	0.910485	1	EPAS1_KB742444.1	32906	14745	0.95139	1.2916	0.047409	1.7906	0.58065	Exon 12
