# Supplementary material for: Convergent evolution on the hypoxia-inducible factor (HIF) pathway genes EGLN1 and EPAS1 in high-altitude ducks
Source: Heredity (Edinb). 2019 Jan 10;122(6):819–32. doi: 10.1038/s41437-018-0173-z (PMC6781116; doi:10.1038/s41437-018-0173-z)
Supplement: Supplementary file 9 — Supplemental figure titels [file 41437_2018_173_MOESM9_ESM.docx]

**Supplemental Figure Information**

**SUPP Figure 1**:

The HIF signaling pathway, and downstream targets. Genes highlighted in red are those which were sequenced through target-enrichment; some members sequenced are paralogs of existing members of this pathway or of downstream targets (in red text). Modified from KEGG database (Kanehisa and Goto 2000) - image used with written permission from Kanehisa Laboratories.

**SUPP Figure 2**:

Nucleotide alignments and protein alignments for the high- and low-altitude populations of both yellow-billed pintail (above) and speckled teal (below) in exon 12 of EPAS1.

**SUPP Figure 3**:

Protein alignments of human and mallard reference sequences for exons 6 and 12 in EPAS1, and exon 2 in EGLN1, showing conservation of protein sequence.
